# Altered platelet function in persistent depressive disorder: a study of density subpopulations and fibrinogen binding

**DOI:** 10.1007/s00702-026-03138-8

**Published:** 2026-03-27

**Authors:** Micha Milovanovic

**Affiliations:** 1 Department of Health, Medicine and Care (HMV), Linköping University, Linköping, Sweden; 2 Department of Health, Medicine and Care (HMV), Faculty of Medicine and Health Sciences, Linköping University, Norrköping, SE-601 74 Sweden

**Keywords:** Persistent depressive disorder, platelets, platelet heterogeneity, platelet activity

## Abstract

Persistent Depressive Disorder (PDD) is a chronic mood disorder linked to psychological and physical health burdens, including increased coronary heart disease (CHD) risk. Emerging evidence suggests platelet activation may represent a shared biological mechanism between PDD and CHD, involving inflammatory and neurovascular pathways. This study aimed to compare platelet activity and reactivity in individuals with PDD and matched controls without psychiatric disorders, using flow cytometric analysis to assess activation markers and platelet subpopulation profiles. Thirty-nine PDD patients receiving SSRI treatment and 103 controls without depressive episodes or other psychiatric disorders were recruited in Sweden. Blood samples were collected and analyzed using flow cytometry and gradient separation techniques. The first sample assessed in vivo platelet activation via fibrinogen binding to glycoprotein Ib across density-separated fractions. The second sample evaluated in vitro platelet reactivity following stimulation with ADP and TRAP-6, with fibrinogen binding as the primary outcome. Platelet counts and mean platelet volume (MPV) were also measured. Results showed no significant differences in platelet distribution across the gradient. However, PDD individuals exhibited significantly higher in vivo platelet-bound fibrinogen in high and normal density fractions (fractions 3–9) and increased platelet reactivity upon agonist stimulation compared to controls (*p* < 0.05). MPV was also significantly elevated in the PDD group. These findings indicate platelet hyperactivation in PDD, potentially contributing to cardiovascular risk. Heightened platelet activation in PDD without comorbid CHD indicates abnormalities intrinsic to depressive pathophysiology. Given platelet activation’s established role in CHD, these findings suggest shared platelet‑driven inflammatory and neurovascular mechanisms linking mood disorders with broader cardiometabolic risk. Further research is needed to clarify these biological links and assess platelet heterogeneity as a potential biomarker for disease progression and therapeutic response in mood and cardiovascular disorders.

## Introduction

Persistent Depressive Disorder (PDD) is a chronic mood disorder defined by a depressed mood lasting at least two years in adults, accompanied by symptoms such as low energy, poor concentration, and hopelessness. Unlike major depressive disorder (MDD), PDD is characterized by its long duration and less severe but persistent symptoms. As outlined in DSM-5, PDD includes both chronic major depression and dysthymia, reflecting the fluctuating yet enduring nature of depressive symptoms. Affecting approximately 2–5% of the population, PDD is linked to significant functional impairment, elevated suicide risk, and frequent comorbidity with anxiety and personality disorders. Early onset and subtle presentations often contribute to underdiagnosis (Substance Abuse and Mental Health Services Administration 2016).

PDD is a chronic mood disorder that exerts substantial influence on both psychological and somatic health domains. It is frequently comorbid with other psychiatric conditions, including anxiety disorders, substance use disorders, and bipolar spectrum disorders. Beyond its psychological burden, PDD is increasingly recognized for its association with adverse physical health outcomes. Individuals with PDD commonly report persistent fatigue and low energy levels, which may reflect underlying neurovegetative dysregulation (Glasner and Eastlick 2024). Chronic pain syndromes, including musculoskeletal and neuropathic pain, are prevalent and may be mediated by shared inflammatory and neurochemical pathways (Rogers et al. 2020). Furthermore, coronary heart disease (CHD) remains a leading cause of morbidity and mortality worldwide, with its global burden continuing to rise annually. While traditional risk factors such as hypertension, hyperlipidemia, diabetes mellitus, smoking, and obesity are well-established contributors, recent epidemiological studies have identified depression, including chronic forms such as PDD, as an independent risk factor for CHD (Lichtman et al. 2014; Pan et al. 2024). The co-occurrence of coronary heart disease (CHD) and depressive disorders, including PDD, substantially contributes to the global healthcare burden, exacerbating morbidity, mortality, and resource utilization (Jha et al. 2019). The underlying mechanisms linking PDD and CHD are multifactorial and complex. Proposed pathways include dysregulation of the hypothalamic-pituitary-adrenal (HPA) axis, autonomic nervous system dysfunction, systemic inflammation, endothelial dysfunction, and notably, platelet activation (Xu et al. 2024).

Platelets, beyond their classical role in hemostasis, are increasingly recognized as mediators of thromboinflammatory and neurovascular processes. Upon activation, platelets undergo morphological changes, redistribute glycoprotein receptors (e.g., GPIb), expose procoagulant phospholipids, and initiate granule release. The GPIIb/IIIa complex, which facilitates fibrinogen binding, undergoes conformational changes detectable via flow cytometry using antibodies such as PAC-1 (Laspa et al. 2025). Recent studies have highlighted that individuals with PDD exhibit enhanced platelet reactivity, which may contribute to the increased cardiovascular risk observed in this population (Izzi et al. 2020; Welt et al. 2004). Platelet activation markers such as soluble CD40L have been found elevated in depressed patients, suggesting a prothrombotic state (Welt et al. 2004).

Platelets also display considerable heterogeneity in terms of density, size, and functional responsiveness. This heterogeneity is increasingly understood to reflect differences in platelet age, granule content, and activation potential (van der Meijden et al. 2019; Veninga 2023) High-density platelets, enriched in α and dense granules, are typically more metabolically active and procoagulant. Conversely, low-density platelets may exhibit altered serotonin storage and release, with implications for neuropsychiatric conditions such as Alzheimer’s disease and fibromyalgia (van der Meijden et al. 2019). Advancements in flow cytometry and single-cell analysis have enabled more precise characterization of platelet subpopulations, revealing distinct phenotypes associated with various pathological states (van der Meijden et al. 2019; Veninga 2023). Our laboratory has previously demonstrated that both high- and low-density platelets can circulate in an activated state, albeit with differing profiles of P-selectin expression and granule content. These findings suggest that platelet heterogeneity may serve as a biomarker for disease activity and therapeutic response (Milovanovic et al. 2003, 2010).

### Aim

The aim of this study was to investigate whether platelet activity and reactivity differed between individuals diagnosed with PDD and a matched control group (CON) without PDD, using flow cytometric analysis of platelet activation markers and subpopulation profiling.

## Materials and methods

### Subjects

This study included two cohorts: individuals diagnosed with PDD and a healthy control group (CON). A total of 39 patients with PDD were recruited from a psychiatric outpatient clinic affiliated with a hospital in Sweden. The mean age of the PDD group was 48 ± 14 years (mean ± standard deviation). All participants met the diagnostic criteria for PDD according to the Diagnostic and Statistical Manual of Mental Disorders, Fourth Edition (DSM-IV). Inclusion criteria required the presence of a persistently depressed mood, occurring for most of the day and on more days than not, as confirmed either by self-report or clinical observation, sustained over a minimum duration of two years. Patients who declined participation or failed to meet diagnostic criteria were excluded. At study entry, all patients were receiving treatment with selective serotonin reuptake inhibitors (SSRIs). In accordance with current Swedish treatment recommendations, SSRIs constitute the first‑line pharmacological option for persistent or moderate‑to‑severe major depressive disorder. This is consistent with both the National Board of Health and Welfare’s clinical guidelines and the recommendations issued by the Swedish Medical Products Agency, which highlight SSRIs as the primary therapeutic intervention owing to their well‑established antidepressant efficacy, comparatively favourable adverse‑effect profile, and substantially lower toxicity in overdose relative to older classes of antidepressant medications.

The control group comprised 103 individuals (mean age 62 ± 8 years) recruited from a primary care centre in Sweden. These participants were attending their routine annual health examination within primary care. As part of standard preventive care, they underwent assessments including blood pressure measurement, fasting glucose, lipid profile, renal function tests, and other routine laboratory investigations in accordance with Swedish primary care guidelines. All control subjects were screened to exclude any current or past depressive episodes or other psychiatric disorders, and written informed consent was obtained from all participants prior to enrolment.

### Laboratory procedures

Venous blood samples were obtained from the antecubital vein of each participant using standard phlebotomy techniques. Two samples were collected into Vacutainer™ tubes (Becton Dickinson, NJ, USA).

*Sample 1:* Platelet Density Separation and in vivo activity.

The first sample (7.5 mL) was anticoagulated with 2.5 mL of a platelet-inhibitory solution* and used for platelet density separation via a linear Percoll™ gradient (GE Healthcare Bio-Sciences AB, Sweden), following established protocols (Järemo 1995; Milovanovic et al. 2003, 2010). Two Percoll™ solutions of different densities (1.09 and 1.04 kg/L) were prepared using components illustrated in Table 1.

**Table 1:** Percoll™ solutions wthit different densities (1.09 and 1.04 kg/L)

PercollTM solutions	1.09 kg/L	1.04 kg/L
Percoll™	32.84 g	8.88 g
H₂O	11,42 g	19.14 g
Platelet-inhibitory solution*	4.50 g	3.00 g

Platelet-inhibitory solution*

*The platelet-inhibitory solution was compiled of equal parts of the components:

* 0.15 mol/L Na₂ citrate and 0.13 mmol/L Na₃EDTA (pH 7.4 at 25°C)* *0.001 g/L prostaglandin E₁ and 1 mL 95% ethanol in H₂O* *2.7 mmol/L theophylline in 0.15 mmol/L TRIS chloride buffer (pH 7.4 at 25°C)*

*(All reagents from Sigma-Aldrich, MO, USA)*

A two-chamber gradient maker (Bio-Rad, CA, USA) was employed to generate linear Percoll™ gradients in 50 mL centrifuge tubes, spanning densities from 1.09 to 1.04 kg/L. The gradient was constructed in three layers:

1. Bottom layer: 7.63 g of the 1.09 kg/L Percoll™ solution.
2. Middle layer: A mixture of 13.08 g of the 1.09 kg/L and 12.48 g of the 1.04 kg/L solutions.
3. Top layer: 10 mL of anticoagulated whole blood, gently layered to avoid disruption of the gradient.

**Fig. 1:**
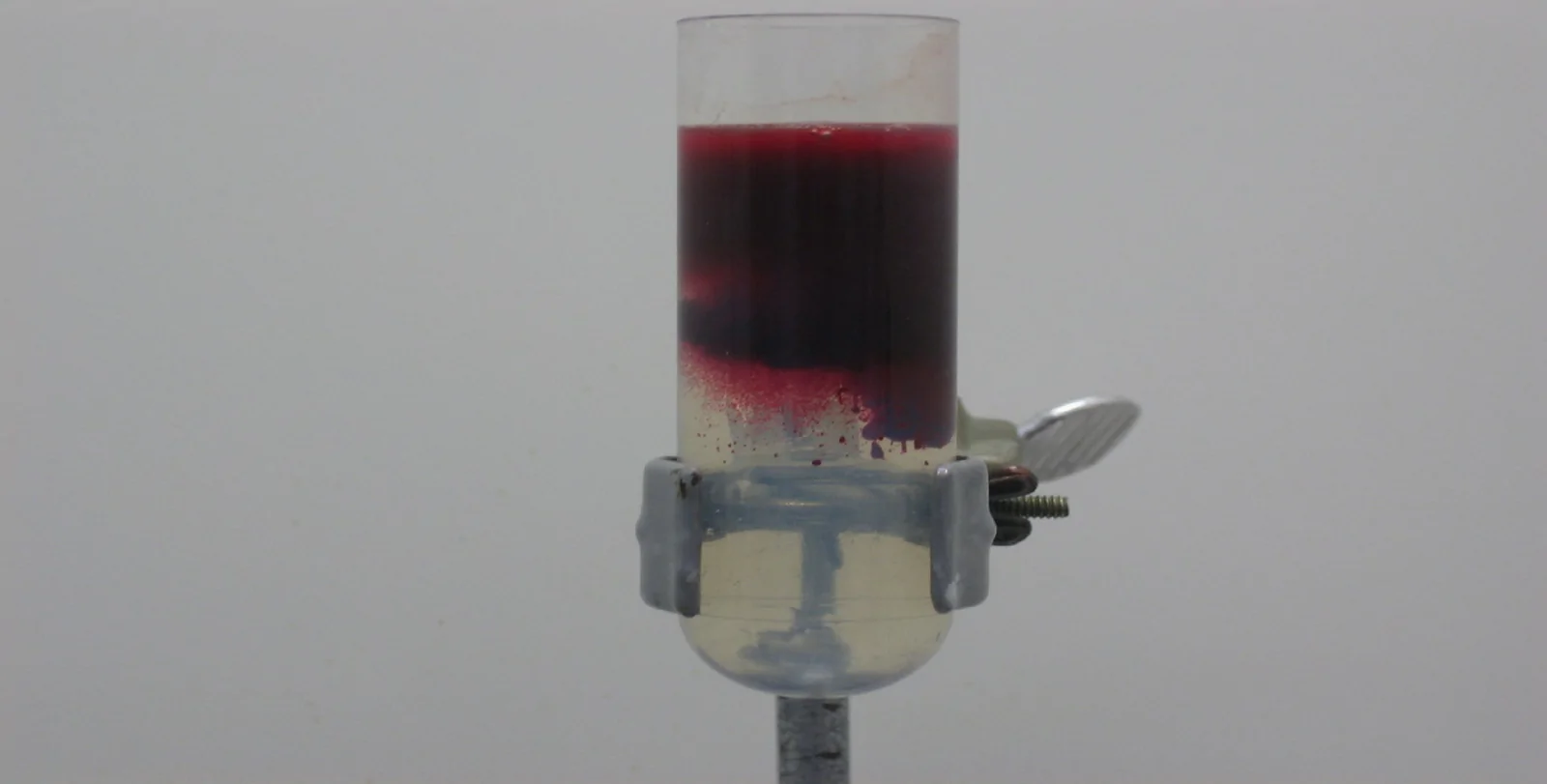
Presents a photograph of the gradient before centrifugation

Centrifugation was performed at 2767 × g for 90 min at room temperature using a swing-out rotor (Fig. 1).

**Fig. 2:**
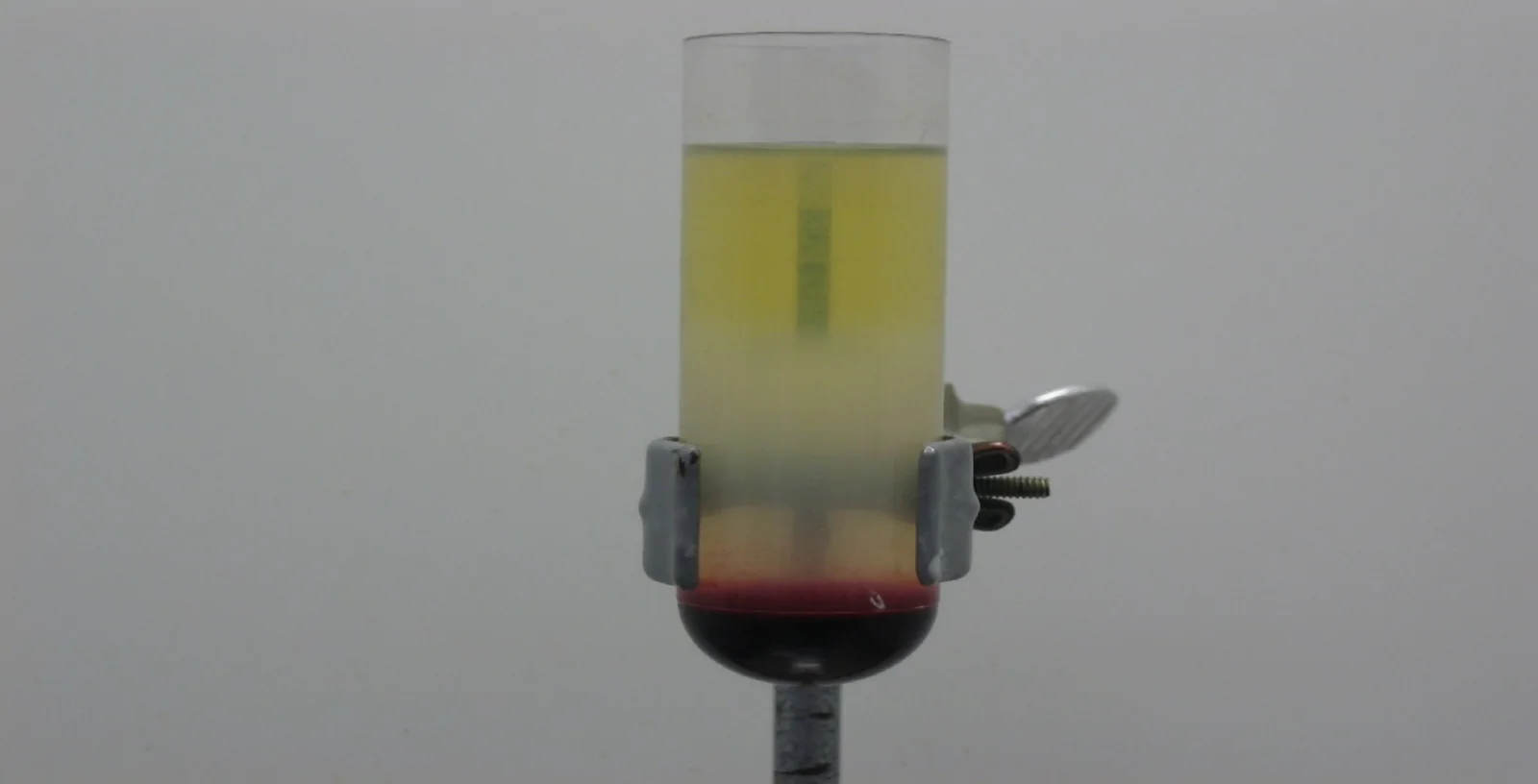
The gradient immediately after centrifugation. The platelet layer is visible in the middle of the tube

**Fig. 3:**
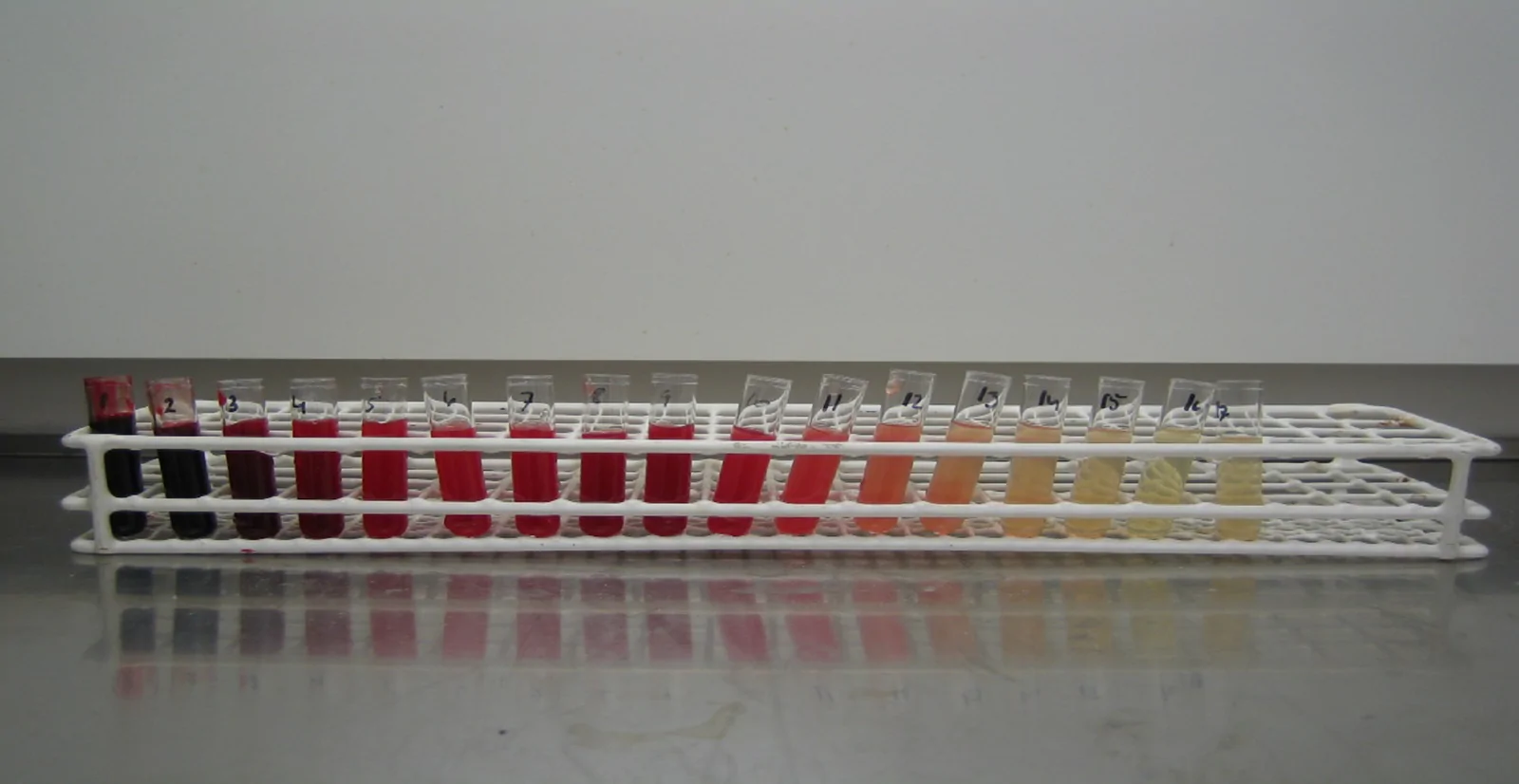
An illustration showing the density fractions after the gradient has been divided into subfractions

Following centrifugation (Fig. 2), the tube was pierced at the bottom with a hot needle, and the gradient was fractionated by gravity into 17 discrete layers, each approximately 2 mL in volume (Fig. 3).

Platelet concentrations in each fraction were quantified using a CELL-DYN 4000 hematology analyzer (Abbott Diagnostics, IL, USA). To assess platelet activation, platelet bound fibrinogen was measured by flow cytometry (EPICS XL MCL™, Beckman Coulter, CA, USA). Platelets were identified phycoerythrin (PE)-conjugated monoclonal antibodies targeting glycoprotein Ib (GPIb; Dako AS, Denmark), and surface bound fibrinogen was detected with a FITC conjugated chicken anti human fibrinogen polyclonal antibody (Biopool AB, Sweden). The FITC‑conjugated anti‑human fibrinogen antibody was supplied as a ready‑to‑use reagent without a specified stock concentration. As this product line did not include an mg/ml value, the antibody was used at a 1:50 volumetric dilution (Lindahl et al. 1992). However, as no agonist was added in this setting, fibrinogen positivity reflects in vivo platelet activation. Instrument settings and fluorescence compensation were verified daily with calibration beads. A structured gating strategy was applied: debris was excluded by FSC and SSC, singlets were selected using FSC‑area versus FSC‑height, and platelets were identified by GPIb positivity. All analyses of fibrinogen binding were then performed within this GPIb‑defined platelet population, with FITC fluorescence used to quantify bound fibrinogen. Negative controls were included to determine background fluorescence and ensure specificity of the signal. This approach provided a clean platelet gate and allowed reliable assessment of fibrinogen binding as a marker of platelet activation.

***Sample 2:*** Platelet reactivity assessment via agonist stimulation.

The second sample (3.5 mL), anticoagulated with 0.5 mL of 0.129 M disodium citrate, was used to assess platelet reactivity following stimulation with agonists. As in sample 1, the same instrument was used, namely the Beckman Coulter EPICS XL‑MCL™ Flow Cytometer (Beckman Coulter, Inc., California, USA). Fluorescent labeling was performed using phycoerythrin (PE)-conjugated monoclonal antibodies directed against glycoprotein Ib (GPIb; Dako AS, Denmark). A FITC‑conjugated chicken anti‑human fibrinogen polyclonal antibody (Biopool AB, Sweden) was used to detect surface‑bound fibrinogen at the same optimized dilution as described for sample 1. In this sample, platelets were stimulated with ADP at concentrations of 1.7 µmol/L and 8.5 µmol/L (Sigma‑Aldrich, MO, USA), as well as with thrombin receptor‑activating peptide (TRAP‑6) at 57 µmol/L and 74 µmol/L (Biotechnology Centre of Oslo, Norway). This procedure simulates in vitro platelet reactivity. The primary readout was the percentage of fibrinogen positive platelets exceeding a threshold defined by the negative control, which contained 10 mmol/L EDTA (Na₂EDTA) prepared in HEPES buffer (4 (2 hydroxyethyl) 1 piperazineethanesulfonic acid). The EDTA solution was prepared using Na₂EDTA (molecular weight 336 g/mol; 3.36 g per liter in deionized water). The HEPES buffer consisted of 137 mmol/L NaCl, 2.7 mmol/L KCl, 1 mmol/L MgCl₂, 5.6 mmol/L glucose, 1 g/L bovine serum albumin, and 20 mmol/L HEPES, adjusted to pH 7.40 (Lindahl et al. 1992). The gating strategy was identical to that used for sample 1.

### Statistical analysis

Statistical analyses were performed using Microsoft Excel^®^, version 12.0.6514.5000. Data are presented as mean ± standard deviation (SD). Group comparisons were conducted using the unpaired Student’s *t-*test. A *p*-value ≤ 0.05 was considered statistically significant.

## Results

The distribution of platelets across the 17 density fractions obtained via Percoll™ gradient centrifugation is summarized in Table 2. No statistically significant differences were observed between the PDD group and the CON group in terms of overall platelet distribution across the gradient layers (*p* > 0.05 i.e. Not Significant for all fractions). These findings suggest that the relative proportion of platelet subpopulations, as defined by density, does not differ markedly between individuals with PDD and healthy controls.

**Table 2:** Platelet density distribution x 10^9^/L in fraction nos. 1–17

Fraction no.	PDD	CON	*p* value
1	8	8	NS
2	15	11	NS
3	11	12	NS
4	15	24	NS
5	29	36	NS
6	57	63	NS
7	82	90	NS
8	139	140	NS
9	184	201	NS
10	137	142	NS
11	49	28	NS
12	21	15	NS
13	12	11	NS
14	9	9	NS
15	8	9	NS
16	9	10	NS
17	12	12	NS

Table 3 illustrates in vivo platelet-bound fibrinogen, representing platelet activity across the density fractions. Patients diagnosed with PDD exhibited significantly elevated levels of surface-bound fibrinogen in fractions corresponding to high and normal density platelets (fractions 3–9), compared to the CON group (*p* < 0.05). These findings suggest an increased in vivo platelet activation state in individuals with PDD, particularly within metabolically active platelet subpopulations.

**Table 3:** In vivo platelet bound fibrinogen (%)

Fraction no.	PDD	CON	*p* value
1	76,14	75,43	NS
2	72,28	69,77	NS
3	68,73	47,11	*p* < 0.05
4	63,72	40,14	*p* < 0.05
5	58,00	32,99	*p* < 0.05
6	54,84	27,32	*p* < 0.05
7	39,81	25,34	*p* < 0.05
8	32,68	19,87	*p* < 0.05
9	27,83	18,54	*p* < 0.05
10	20,71	17,73	NS
11	18,63	20,56	NS
12	25,51	27,14	NS
13	30,58	32,09	NS
14	35,27	36,29	NS
15	33,57	37,32	NS
16	35,87	38,21	NS
17	33,50	35,72	NS

The second sample, anticoagulated with 0.5 mL of 0.129 M disodium citrate, was used to assess in vitro platelet reactivity. This was evaluated by measuring fibrinogen binding following stimulation with ADP (1.7 and 8.5 µmol/L) and TRAP‑6 (57 and 74 µmol/L), and the corresponding results are presented in Table 4.The PDD group demonstrated significantly greater platelet reactivity than controls across all agonist conditions (*p* < 0.05).

**Table 4:** In vitro platelet bound fibrinogen (%)

Stimulus	PDD	CON	*p* value
ADP 1,7 µmol/L	9,44	5,11	*p* < 0.05
ADP 1,7 µmol/L	8,69	4,98	*p* < 0.05
ADP 8,5 µmol/L	19,83	12,11	*p* < 0.05
ADP 8,5 µmol/L	19,92	13,03	*p* < 0.05
TRAP-6 54 µmol/L	24,91	16,24	*p* < 0.05
TRAP-6 54 µmol/L	25,12	15,97	*p* < 0.05
TRAP-6 74 µmol/L	29,14	17,32	*p* < 0.05
TRAP-6 74 µmol/L	28,47	15,11	*p* < 0.05

Additionally, mean platelet volume (MPV) was significantly higher in the PDD group compared to controls (*p* < 0.05; data not shown), indicating larger platelet size among depressed individuals.

## Discussion

To our knowledge, this is the first study to investigate platelet density subpopulations in individuals with PDD in relation to platelet-bound fibrinogen. The findings reveal that patients with PDD exhibit both elevated in vivo platelet activity and increased in vitro platelet reactivity compared to healthy controls.

No significant differences in platelet subpopulation distribution, as defined by density, were observed between individuals with PDD and CON. To date, there is no published evidence indicating that PDD is associated with deviations in total platelet count, suggesting that functional platelet parameters may be more relevant biomarkers in this population.

The significant differences observed in platelet activation across density fractions (Table 3). suggest that platelets of both high and normal density are more activated in PDD. Furthermore, enhanced reactivity to platelet agonists (Table 4) supports the notion of a hyperreactive platelet phenotype in depression. These findings mirror patterns previously described in CHD, where activated platelets contribute to atherosclerotic plaque formation and disease progression (Badimon et al. 2012; Willoughby et al. 2002). Depression has been consistently associated with an increased risk of CHD, including higher incidence of myocardial infarction and cardiovascular mortality (Carney and Freedland 2008; Frasure-Smith and Lesperance 2005; Van Melle et al. 2004). Our results support the hypothesis that PDD and CHD may share common pathophysiological mechanisms, potentially mediated by platelet activation. It is also plausible that platelets may influence neuronal function. Alterations in neuronal activity and structure have been observed in mood disorders, and previous research has suggested a link between platelet activity and pain perception, another symptom common to both PDD and CHD (Milovanovic et al. 2016). These observations point to several potential shared mechanisms, including inflammatory pathways, that warrant further investigation.

Mean platelet volume (MPV), a surrogate marker of platelet activation, has been proposed as a predictive biomarker in cardiometabolic diseases. Elevated MPV has been associated with acute myocardial infarction (Cameron et al. 1983), coronary atherosclerosis (Martin et al. 1991) and stroke risk (Greisenegger et al. 2004). In this study, PDD patients exhibited significantly higher MPV values, further supporting the association between depression and altered platelet function.

The interpretation of platelet hyperreactivity in PDD must also take into account the well‑established effects SSRIs on platelet biology. SSRIs inhibit the serotonin transporter (SERT) on platelets, leading to depletion of intraplatelet serotonin and attenuation of serotonin‑dependent amplification of aggregation. Consistent with this mechanism, several experimental studies have demonstrated reduced platelet activation and diminished responsiveness to agonists in SSRI‑treated individuals (McCloskey et al. 2008; Tseng et al. 2013; Grech et al. 2022a, 2022b), and mechanistic work further shows that agents such as citalopram and fluoxetine modulate downstream signalling pathways and inhibit serotonin‑potentiated aggregation independently of serotonin depletion (Roweth 2018; Bampalis et al. 2010). Against this background, the increased fibrinogen binding both at baseline and following agonist stimulation observed in our SSRI‑treated PDD cohort appears counterintuitive.

However, depression itself has repeatedly been associated with heightened platelet activation (Morel‑Kopp et al. 2009), making it essential to distinguish disease‑related alterations from pharmacological effects. Several factors may explain the apparent discrepancy between the expected inhibitory influence of SSRIs and the hyperreactive phenotype observed in our study. First, most previous investigations have examined whole platelet populations rather than density‑defined subfractions, potentially obscuring functional heterogeneity within the platelet compartment. Second, elevated baseline activation in PDD may reflect underlying inflammatory or metabolic disturbances intrinsic to the disorder rather than SSRI effects per se (Dowlati et al. 2010; Lopresti et al. 2014). Third, the higher MPV observed in the PDD group suggests a shift towards larger and intrinsically more reactive platelets, which may counterbalance or override SSRI‑mediated inhibition. Finally, inter‑individual variability in SSRI dosage, treatment duration, and pharmacodynamic response may contribute to heterogeneous platelet phenotypes (Serebruany et al. 2005). Taken together, these considerations suggest that SSRI‑mediated inhibition of platelet activation, although mechanistically robust, may be insufficient to counteract the heightened pro‑activatory milieu associated with PDD, ultimately giving rise to the net hyperreactive platelet phenotype observed in our study.

Elevated platelet activation observed in patients with depression may contribute to their increased cardiovascular risk. Considering the findings from this study, it is plausible that a more intensive SSRI regimen may be required to achieve sufficient attenuation of platelet activation, thereby potentially reducing cardiovascular events in individuals with established heart disease and concomitant PDD. Our platelet data derive primarily from cohorts with PDD and do not specifically include patients with CHD and comorbid depression. To evaluate whether SSRIs confer cardiovascular benefit through modulation of platelet function, adequately powered, prospective, randomised trials in CHD populations with depression are required. Such trials should prespecify clinical cardiovascular endpoints, incorporate standardised and validated biomarkers of platelet activation, include stratification for key confounders (for example, antiplatelet therapy and cardiovascular risk profile), and ensure sufficient follow‑up to assess effects on morbidity and mortality.

The clinical implications of platelet heterogeneity remain uncertain. The origin and significance of the observed differences in platelet density and activation require further elucidation. Larger studies are needed to confirm these findings and to better understand the role of platelet biology in the pathogenesis of depression and its cardiovascular comorbidities.

### Limitation of the study

One notable limitation of this study is the age disparity between the PDD and control groups, with the control group having a significantly higher mean age (62 ± 8 years) compared to the PDD group (48 ± 14 years). Age may influence platelet function and cardiovascular risk factors, which may confound the observed differences in platelet activation and reactivity. This discrepancy could limit the generalizability of the findings and complicate the interpretation of whether platelet hyperactivity is attributable to PDD itself or age-related physiological changes. Additionally, the sample size of the PDD group (*n* = 39) is relatively small compared to the control group (*n* = 103), which may affect the statistical power and robustness of the results. Recruitment from a single psychiatric outpatient clinic may also introduce selection bias, limiting the representativeness of the PDD cohort. A further important limitation of the present study is the lack of individual‑level data on antidepressant treatment. Although all participants with PDD were recorded as receiving SSRIs at study entry, the dataset did not capture the specific SSRI agent, dose, treatment duration, initiation date, adherence or any changes in therapy during follow‑up. Consequently, the study were unable to investigate agent‑specific or dose-response relationships, to assess the influence of treatment duration or adherence on platelet measures, or to explore potential interactions between particular SSRIs and concomitant medications. This limitation constrains causal inference regarding whether the observed differences in platelet activation are attributable to SSRI exposure per se, to heterogeneity between individual agents, or to unmeasured confounding by indication. It also precludes sensitivity analyses that might determine whether certain drugs within the SSRI class exert stronger modulatory effects on platelet function. Future studies should therefore prospectively record detailed pharmacotherapy data (agent, dose, timing, adherence and, where feasible, plasma concentrations) and incorporate stratified or pharmacoepidemiological analyses to disentangle agent‑specific effects from class effects and residual confounding.

Finally, while the study excluded individuals with comorbid CHD, it did not account for other potential confounding factors such as medication use (e.g., SSRIs), lifestyle variables (e.g., smoking, diet, physical activity), or inflammatory markers, all of which could influence platelet function.

## Conclusion

This study provides novel insights into platelet biology in PDD, including the first characterization of platelet density subpopulations in relation to platelet‑bound fibrinogen. Patients with PDD demonstrated a hyperreactive platelet phenotype, with elevated in vivo activation and enhanced in vitro responsiveness to agonists. Although platelet density distribution did not differ between groups, significant activation across density subpopulations indicates functional heterogeneity with potential pathophysiological relevance. Together with increased MPV and unchanged total platelet counts, these findings highlight functional platelet alterations as potential biomarkers in PDD. Importantly, the observation that heightened platelet activation occurs in the absence of comorbid CHD suggests that these abnormalities are intrinsic to depressive pathophysiology rather than secondary to cardiovascular disease. The results reinforce the hypothesis of shared mechanisms between PDD and CHD, potentially mediated by platelet‑driven inflammatory and neurovascular pathways. Such mechanisms may represent a convergent biological axis linking mood disorders with cardiometabolic risk. Given the widespread use of SSRIs and their known inhibitory effects on platelet function, future work should determine whether pharmacological modulation can meaningfully alter this hyperreactive phenotype and whether such modulation translates into measurable cardiovascular benefit. Larger, longitudinal studies are warranted to clarify the clinical implications of platelet heterogeneity and its role in the neurovascular interface of mood disorders.

## Data Availability

The datasets used and/or analyzed during the current study are available from the corresponding author on reasonable request.
